# Influence of Noise Correction on Intra- and Inter-Subject Variability of Quantitative Metrics in Diffusion Kurtosis Imaging

**DOI:** 10.1371/journal.pone.0094531

**Published:** 2014-04-10

**Authors:** Elodie D. André, Farida Grinberg, Ezequiel Farrher, Ivan I. Maximov, N. Jon Shah, Christelle Meyer, Mathieu Jaspar, Vincenzo Muto, Christophe Phillips, Evelyne Balteau

**Affiliations:** 1 Cyclotron Research Centre, University of Liège, Liège, Belgium; 2 Institute of Neuroscience and Medicine - 4, Juelich, Germany; 3 Department of Neurology, Faculty of Medicine, Jülich Aachen Research Alliance, RWTH Aachen University, Aachen, Germany; 4 Department of Electrical Engineering and Computer Science, University of Liège, Liège, Belgium; INSERM U894, Centre de Psychiatrie et Neurosciences, Hopital Sainte-Anne and Université Paris 5, France

## Abstract

Diffusion kurtosis imaging (DKI) is a promising extension of diffusion tensor imaging, giving new insights into the white matter microstructure and providing new biomarkers. Given the rapidly increasing number of studies, DKI has a potential to establish itself as a valuable tool in brain diagnostics. However, to become a routine procedure, DKI still needs to be improved in terms of robustness, reliability, and reproducibility. As it requires acquisitions at higher diffusion weightings, results are more affected by noise than in diffusion tensor imaging. The lack of standard procedures for post-processing, especially for noise correction, might become a significant obstacle for the use of DKI in clinical routine limiting its application. We considered two noise correction schemes accounting for the noise properties of multichannel phased-array coils, in order to improve the data quality at signal-to-noise ratio (SNR) typical for DKI. The SNR dependence of estimated DKI metrics such as mean kurtosis (MK), mean diffusivity (MD) and fractional anisotropy (FA) is investigated for these noise correction approaches in Monte Carlo simulations and in *in vivo* human studies. The intra-subject reproducibility is investigated in a single subject study by varying the SNR level and SNR spatial distribution. Then the impact of the noise correction on inter-subject variability is evaluated in a homogeneous sample of 25 healthy volunteers. Results show a strong impact of noise correction on the MK estimate, while the estimation of FA and MD was affected to a lesser extent. Both intra- and inter-subject SNR-related variability of the MK estimate is considerably reduced after correction for the noise bias, providing more accurate and reproducible measures. In this work, we have proposed a straightforward method that improves accuracy of DKI metrics. This should contribute to standardization of DKI applications in clinical studies making valuable inferences in group analysis and longitudinal studies.

## Introduction

Diffusion weighted (DW) magnetic resonance imaging (MRI) [1] and diffusion tensor imaging (DTI) [2] are nowadays widely applied imaging modalities allowing one to study the complexity of neuronal networks of axons and to characterize the microstructural properties of brain tissues on the length scale of cellular size [3]. DTI focuses on the study of white matter (WM) structure and provides important information about the tissue anisotropy characterizing neuronal fiber tracks. In this approach, the attenuation of the DW signal is approximated by a mono-exponential function valid for rather low diffusion weightings (*b*-values), typically not exceeding 1000 s/mm^2^) [4]–[6]. At higher *b*-values, the deviations from the mono-exponential decay occur, related to the complexity of the brain tissue microstructure hindering and restricting the diffusion of water molecules at different length scales [7] (e.g. cell membranes of varying permeability, organelles with a wide range of sizes, shapes and packing densities). In turn, the level of complexity may shed light on microstructural changes or damages in both the healthy and pathological brain. Recently, diffusion kurtosis imaging (DKI) has been proposed by [8], [9] to capture changes related to patterns of non-Gaussian diffusion within clinically reasonable acquisition time. It is an extension to DTI, making use of the second term in the Taylor series expansion of the logarithm of the DW signal according to:
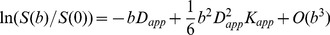
(1)where *b* denotes the strength of the diffusion weighting and D_app_ and K_app_ are the apparent diffusion coefficient and excess diffusion kurtosis, respectively. The conventional DTI parameters such as fractional anisotropy (FA) and mean diffusivity (MD) are estimated as well as the mean kurtosis (MK). Kurtosis allows one to quantify the degree of diffusion non-Gaussianity and provides an empirical measure of the level of heterogeneity of WM tissues [10]. Within the frame of WM model, kurtosis metrics were related to the axonal water fraction [11], [12] and to the restricted volume fraction using the CHARMED model [13]. Furthermore, as mean kurtosis (MK) does not require anisotropic tissue organization, gray matter (GM) microstructure can also be investigated [10], [14]. In recent years, the interest for DKI has been continuously growing and MK has shown great potential as a biomarker to detect tissue abnormalities, being more sensitive to changes than classical DTI metrics. Promising results have been reported in the study of ischemic stroke in both human [15] and animal models [16], brain gliomas [17] and epilepsy [18]. DKI might thus become a useful clinical tool in the coming years.

However, the processing part is still not well established and there is no standard methodology, which makes it more difficult to use clinically. The direct impact of acquisition parameters or data processing has not been sufficiently studied, especially related to intra- and inter-subject variability. However this information is very valuable for clinical studies, in particular, for quantifying the changes on the individual and group levels, and assessing their significance in longitudinal studies. Therefore, the study of inter-subject variability of diffusion metrics in general has recently gained momentum [19]–[21]. To our knowledge, only two studies have investigated inter-subject variability in DKI: one for improving the study design in terms of statistical power [22] and the second to provide some reference values [23]. None of them included noise bias corrections. It is thus important to identify changes in kurtosis parameter variability in relation to the acquisition setup and data processing. In particular, one of the difficulties of DKI in comparison to DTI is the need of DW signal at higher diffusion weighting coefficients (*b*-values ≤2500–3000 s/mm^2^). For conventional DTI (*b*-values ≤1000 s/mm^2^), SNR is still relatively high and the impact of noise on the estimation of parameters such as fractional anisotropy (FA) or mean diffusivity (MD) is relatively small [24]. However, as diffusion weightings becomes larger the signal drops rapidly, so is the SNR and the signal can easily reach the noise floor [25]. This is particularly true in regions experiencing fast signal decay because of the free diffusion of the molecules (cerebrospinal fluid (CSF)) or because of the high degree of directionality along a specific direction (e.g. along the fibers in the WM). When the signal is about or below the noise floor, the noise introduces a significant bias artificially enhancing the measured signal intensity [26]. The noise is then interpreted as true signal and, if not corrected, leads to an overestimation of kurtosis [27]–[29]. Thus in clinical applications, where the number of repetitions is limited by acquisition time, the low SNR and resulting noise bias can strongly affect the reliability and sensitivity of the diffusion experiment. For diagnosis purposes, as well as to derive medical inferences on the pathological alterations of the brain tissue, the accuracy and reproducibility of the estimated diffusion metrics are essential, which require to account or correct for noise bias.

The characterization of noise in MRI is challenging, especially with the introduction of multiple receiver coils and parallel imaging techniques. In order to avoid phase artefacts, magnitude images are generally preferred to complex images [30]. Both real and imaginary parts of the complex signal recorded by each channel are assumed to be Gaussian-distributed. For a single-channel acquisition, the magnitude reconstruction provides an image whose signal is Rician-distributed while the background noise is Rayleigh-distributed [31]. Nowadays, multichannel receiver coils are routinely used and preferred to quadrature receiver coils, providing higher SNR and reducing the acquisition time and geometric distortions thanks to parallel imaging techniques [32]. The noise properties are influenced by the parallel imaging technique used as well as by the reconstruction filters applied. A review of the noise characteristics under these different configurations can be found in [33]. Data acquired with multichannel phased-array coils and images reconstructed as the root of the sum of squares (SoS) of the complex images of each channel exhibit a signal following a noncentral chi distribution [34]. The background noise, on the other hand, is central chi distributed and can generally be assumed spatially invariant [34]. With advanced parallel imaging methods, the noise distribution becomes spatially dependent, and the signal properties require more complex modelling [27], [35]–[37].

Two main approaches to correct for the noise bias have been described previously. The first approach is based on the correction of the magnitude images prior to model fitting [31], [38]–[42] while the second approach is accounting for the noise bias in the model estimation procedure itself [29], [43]. Both approaches require the estimation of the underlying Gaussian noise standard deviation. For that purpose, different methods using either image background areas or the image object itself have been reviewed in [44]. In the case of DKI, only the second approach has been investigated [26], [29]. The impact of noise, both thermal and physiological, on diffusion metrics has been studied previously for different non-Gaussian models [26], in the case of Rician noise distribution only. The error in estimating DKI derived metrics has been shown to increase as SNR decreases [45], and estimators accounting for the noise bias have been shown to provide more accurate results [27], [28]. Despite noise has been clearly shown to influence the results in DKI, noise correction is not systematically applied. Recently, a number of DKI studies have investigated different aspects such as reproducibility [23], sample size and statistical power [22], the choice of gradient directions and *b*-values [46] or fast acquisition method [47]. However, the impact of noise was not investigated and no correction was applied in these studies.

In this work, we investigate the influence of noise correction on the estimation of DTI and DKI metrics, such as MD, FA or MK, in human data *in vivo* and their dependence on SNR. Two noise correction methods based on the first and second moments of the noncentral chi distribution [34] have been applied and compared with non-corrected data. Noise level was estimated prior model fitting, in order to fit the data to an estimated noise-free signal, and improve accuracy and precision of the diffusion model estimators. The performance of these methods was compared to the maximum likelihood (ML) estimator [27] using simulations and one real data set. The intra-subject reproducibility of the DTI and DKI parameters estimation as a function of SNR is investigated in a single subject experiment. SNR was manipulated by repositioning the head of the subject within the multichannel head coil, taking advantage of the spatially varying sensitivity of each coil in the array, and by varying the spatial resolution. In a second experiment, inter-subject variability of DTI and DKI parameters was investigated in a group of 25 healthy volunteers to provide insight on the suitability and reliability of DTI and DKI metrics for group comparison in clinical studies. Both intra- and inter-subject variability of these metrics were compared in relation to SNR level and correction schemes. We hypothesize that noise correction reduces spurious intra- and inter-subject variability of the estimated parameters, providing more accurate and reproducible biomarkers. We also provide a list of parameter values and variability in typical regions of the brain.

## Methods

### Theory of signal distribution

In most diffusion MRI experiments, the image is reconstructed as the magnitude of the complex image in order to avoid phase shifts artifacts [30], [48]. With multichannel receive coils and SoS combination of the complex images from each of the coils, the measured signal follows a noncentral chi distribution [34] which is expressed as follows:
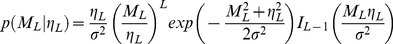
(2)where *L* is the number of coils, *M_L_* is the measured signal, η*_L_* is the signal in the absence of noise (the “true” signal), σ is the standard deviation of the noise and *I_L-1_* is the modified (L-1)^th^ order Bessel function of the first kind. The analytical expressions of the first and second moments of the noncentral chi distribution are given by [34]:
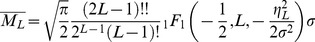
(3)and 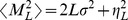
(4)respectively, where _1_ *F*_1_ is the confluent hypergeometric function and (2*L*-1)!!  = 1*3*5*…*(2*L*-1). In the absence of signal (η_L_ = 0), the background noise follows a central chi distribution [34]: 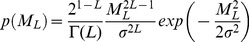
(5)


The first moment of the central chi distribution is non-zero and proportional to σ. As a result, in low SNR voxels, the measured signal *M_L_* is overestimated, affecting the estimation of the DTI and DKI parameters. In order to avoid this noise bias, *η_L_* should be estimated based on accurate estimates of σ and *M_L_* and the analytical expressions given in Eq. (3) and Eq. (4).

### Data acquisition

DW experiments were performed on a head only 3T MRI system (Allegra, Siemens) using an 8-channel receive head coil. DW images were acquired with a twice-refocused spin-echo diffusion sequence. Gradients were allowed their maximum value (40 mT/m) and slew rate (400 T/m/s). Data were reconstructed using SoS reconstruction with equal weights. Two experiments were carried out. For both of them, DW images were acquired along 60 non-coplanar directions at each *b*≠0. For motion correction purpose (see section 2.4 on data processing), twelve non-DW images interleaved with the DW images were acquired. The acquisition time was about 16 minutes for one session.

#### Experiment 1: Intra-subject inter-session variability

The first experiment investigates the intra-subject variability of the DKI parameters as a function of SNR and relies on the assumption that for a single subject, the estimation of the DKI parameters should be reproducible over multiple sessions and be SNR independent. The SNR was manipulated either by repositioning the head of the subject within the head coil, over several repetitions of the same measurement, taking advantage of the spatially varying sensitivity of each coil in the array (Protocol 1a), or varying the spatial resolution (Protocol 1b), see Table 1 for the acquisition parameters of Protocols 1a and 1b. Protocol 1a was repeated five times on the same volunteer (27 years old female) for different head positions. Due to the spatially varying sensitivity of the coil array and the different positions of the head relatively to each coil element, the SNR was spatially dependent, and its spatial distribution varied from one session to the next. As a result, the SNR in a given brain area varied from one session to the next. In the following, the experiments related to Protocol 1a for different head positions will be referred to as P1 (center of the coil), P2 (shift up), P3 (shift down), P4 (shift to the right) and P5 (shift to the left). In Protocol 1b, data were acquired on the same volunteer with centered head position and larger voxel size (3×3×3 mm^3^ instead of 2.4×2.4×2.4 mm^3^) in order to reach a global 2-fold increase in SNR. This higher SNR protocol is expected to show moderate noise correction effects as compared to lower SNR situations. In the following, this protocol will be referred to as P6. The variations in the acquisition parameters including TE, TR, matrix and voxel sizes between protocols affect the SNR but should not affect the reproducibility of diffusion parameter estimation after noise correction is applied, which is at the heart of our study.

**Table 1:** Summary of the acquisition parameters for each protocol.

Parameters	Protocol 1a	Protocol 1b	Protocol 2
Number of repetitions	1	1	3
*b*-value (s/mm^2^)	0/1000/2500	0/1000/2500	0/1000/2800
TR (ms)	7400	6800	7400
TE (ms)	91	88	89
FoV (mm)	211	192	192
Number of slices	54	44	58
Matrix size	88×88	64×64	96×96
Voxel size (mm^3^)	2.4×2.4×2.4	3×3×3	2.2×2.2×2.2

#### Experiment 2: Inter-subject variability

Protocol 2 (see Table 1 for the acquisition parameters) was acquired on 25 healthy male volunteers. In order to reduce the genuine inter-subject variability in the population, data sets were selected from a homogeneous population consisting of male volunteers recruited for an ongoing study with the following criteria: age range, 18–30 years old (mean 23±3); non smokers with no history of neurological or psychological diseases and no medication or drug abuse. The amplitude and spatial distribution of the inter-subject variability was studied as a function of the noise correction procedure and SNR spatial distribution.

#### Ethics Statement

The experimental procedures received approval from the Ethics Committee of the University of Liege and signed informed consent was obtained from all participants prior the beginning of the experiment.

### Noise estimation and magnitude image correction

#### Noise estimation

The methods developed here to correct DW images for noise bias require an estimation of the noise standard deviation, σ. The estimation was performed either from a noise image (acquired with the radio-frequency (RF) transmit amplitude set to zero for all RF pulses) when available (Experiment 1) or from voxels extracted from the background of the DW images (Experiment 2). In the latter case, a mask was created by automatically thresholding the mean non-DW image, excluding both signal and Nyquist ghost voxels. The standard deviation was estimated on the images (noise images or DW images) prior to any processing using the following expression [34]:
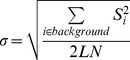
(6)where *S*_i_ is the measured signal for each voxel in the background area, *N* is the total number of voxels and *L* is the number of coils. The validity of Eq. (6) was confirmed in our experimental setup by inspecting the noise distribution in the acquired noise images. The expression given in Eq. (5) (with *L* = 8) closely fitted the histogram of measured noise voxel intensities, with a standard deviation closely matching the value calculated from Eq. (6) (data not shown). Moreover, the difference observed between the standard deviation estimated on the noise images and the standard deviation estimated on the background of the images in Experiment 1 did not exceed 3% when using DW images for background estimation and 6% when using non-DW images. This range of error has no significant impact on the noise correction procedure and the background noise estimation can therefore be used reliably when no noise image is available.

In addition, the noise standard deviation is used to estimate the apparent local SNR in the images, calculated voxel-by-voxel as the mean signal of the non-DW images (prior noise correction) divided by the standard deviation of the noise.

#### Non local mean filtering

Prior to noise correction, a nonlocal mean filter (BM4D) [49] was optionally applied to the data. This filter provides a better estimation of the first and second moments of the measured magnitude, while preserving fine structures and details of the images. Its effect on the estimation of diffusion and kurtosis parameters was investigated at the individual and group level.

#### First moment correction

This method, in the following referred to as M1, is based on the first moment 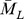 of the noncentral chi distributed signal (Eq. (3)). As this equation has no analytical solution, a look-up table was used to retrieve *η_L_* using linear interpolation. After realignment of the DW images (see Section 2.4), the look-up table of 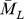 versus *η_L_* was built for each individual image, using the estimated standard deviation 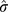 and values of 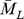 between the noise floor and the maximum measured intensity in the image: small steps were used to guarantee a good accuracy. The noise floor is the minimum value taken by 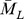, corresponding to *η_L_* = 0. For example with *N* = 8, the noise floor is equal to 3.94*σ. For each pixel, the magnitude of the measured signal (after non local mean filtering if applied) was used as an estimate of the first moment 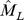 while the true signal estimate 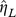 was calculated by interpolation of the look-up table. The corrected signal intensity 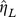 was set to zero whenever the measured intensity 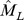 was below the noise floor.

#### Power image correction

The second method, M2, is based on the expression of the second moment (Eq. (4)). This method was first introduced for single channel acquisitions and Gaussian signal distribution [38]. The same approach is used here in the case of multichannel receiver coil and noncentral chi distribution. The second raw moment of the signal distribution 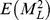 is approximated with the square of the measured signal 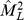 (power image) after non-local mean filtering (if applied), as described in [38]. Then, the correction is applied to the power image: 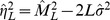 where 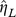
is the true signal amplitude estimate, 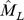
is the estimate of the first moment of the measured signal, *L* is the number of coils, and 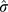 is the estimate of the noise standard deviation. When the measured signal intensity is below the noise floor, the squared true signal amplitude estimate is negative, leading to imaginary numbers in the corrected magnitude image. In such cases the corrected signal intensity 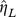 was set to zero.

### Data processing

In all protocols, non-DW images were first realigned with rigid body transformation using SPM8 (Wellcome Trust Centre for Neuroimaging, UCL, UK) to correct for motion, for each individual session. Movements between two non-DW images were interpolated and transformations were applied to the corresponding DW images. The diffusion directions were rotated accordingly [50]. The non-local mean filter was optionally applied and images were optionally corrected for noise with the two correction schemes described above. In total, six different procedures are compared: (a) no correction (NC), (b) non-local mean filtering only (BM4D+NC), (c) first moment method (M1), (d) non-local mean filtering and M1 (BM4D+M1), (e) second moment method (M2), (f) non-local mean filtering and M2 (BM4D+M2).

The logarithm of the normalized signal intensities were fitted to its cumulant expansion truncated at its second order in b, as described in the introduction (Eq. (1)), for each diffusion direction, on a voxel-by-voxel basis, using a nonlinear least square algorithm. Then, the diffusion tensors are estimated by solving a linear system for the tensor components [2] and diagonalized. In a similar fashion, kurtosis tensors were estimated voxel-by-voxel [8]. The kurtosis tensors were transformed from the laboratory coordinate system to a coordinate system defined by the three eigenvectors of the diffusion tensor [51]. Conventional DTI (FA and MD) as well as DKI metrics [10] were evaluated. Among three conventional kurtosis metrics (axial, radial, and mean), we focused our presentation on MK as the most representative and frequently used one. MK was calculated as the averaged apparent kurtosis (evaluated from the kurtosis tensor) over the unit sphere, as described by equation (55) in [10]. In addition, one data set (protocol 1a, P1) was analysed using ML estimator [27]. The original script of this estimator was provided to us by J. Veraart [27].

#### Experiment 1

In order to compare the different sessions, non-DW images were realigned in the image space of the first session and the same spatial transformations were applied to the parameter maps. Few outlier voxels with extremely high fitted values of MK were reassigned with the averaged neighbouring values. Region of interest (ROI) analysis was performed on eight independent regions. These regions were delineated in six different WM and two GM areas using the Harvard-Oxford subcortical structural atlas and the JHU white-matter tractography atlas available in FSL (Analysis Group, FMRIB, Oxford, UK [52]): Temporal Lobe (TL), Internal Capsule (IC), Anterior Corona Radiata (ACR) and the Globus Pallidus (GP), both left and right. MK values from these ROIs were extracted and compared. Pearson's linear correlation tests were performed to determine if the mean MK was significantly correlated or not to SNR for each ROI and each correction separately. SNR maps were derived for each session as described in section 2.3.1. For protocol 1a, z-score analysis was also performed voxel-by-voxel for each correction scheme to evaluate the deviation of each individual MK measure from the average MK map for the six correction procedures.

#### Experiment 2

For group analysis, data from the 25 subjects were normalized to MNI spaces. The mean non-DW images were individually segmented and warped into MNI space using the new segment tool in SPM8 (Wellcome Trust Centre for Neuroimaging, UCL, UK). The same non-linear transformations were applied to the scalar parameter maps. For MD, FA and MK maps respectively, standard deviation maps across the group were calculated for each noise correction scheme and histograms of the MD, FA and MK maps averaged over the 25 subjects were also calculated. GM and WM histograms of MK maps were calculated using corresponding masks. These masks were created using FA maps thresholded at <0.25 for GM and >0.25 for WM. ROI analysis using the ROIs described above (Experiment 1) was also performed on each of the 25 subjects, and the ROI mean and standard deviation of MK over the 25 subjects were calculated for each noise correction scheme and compared.

### Simulations

In addition to the real data acquisitions, an extra experiment using a simulated phantom was performed. The goal is to validate our noise correction methods with Monte-Carlo simulations and to compare the results with those obtained with the ML estimator, as it is an unbiased estimator [27]. A full diffusion kurtosis tensor was simulated, using 60 directions and 3 *b*-values (0,1000,2500 s/mm^2^). FA was set to 0.7606, MD to 0.9487×10^−3^ mm^2^/s and MK to 0.9662. These are typical white matter values. Zero-mean Gaussian noise was added to both real and imaginary part of the noise free signals for each coil to get noisy synthetic data signals. The composite magnitude image was then obtained with the online parallel MRI noisy phantom simulator available online (http://www.lpi.tel.uva.es/~santi/) using the SoS method, assuming no noise correlations for 50×50 voxels image, corresponding to a total of 2500 trials. The mean and mean square error (MSE) of FA, MD and MK were calculated for the three methods (M1, M2 and ML) and compared in order to assess their performances.

## Results

### Simulations

**Figure 1:**
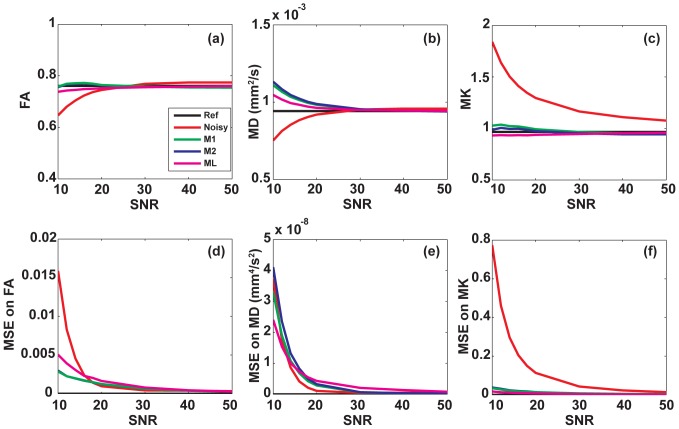
Monte-Carlo simulations. Averaged values of (a) FA, (b) MD, (c) MK over 2500 trials and their corresponding mean square errors (d–f) for different estimators. The results are shown for the uncorrected noisy signal (red), and for the signal corrected by M1 (green), M2 (blue) and ML (magenta). The black line indicates the reference value.

Simulation results are shown in Figure 1. The mean FA, MD and MK values are plotted as a function of SNR. There is a clear deviation from the reference when no noise correction is applied, with an underestimation of FA and MD up to SNR<25 and an overestimation of MK up to SNR = 50. However this bias is considerably reduced when corrections are applied, even for relatively low SNR. M1 and M2 corrections lead to similar results as ML. For the intra- and inter-subject variability study, we thus chose to skip ML estimator, as it is computationally consuming and proceeded only with M1 and M2.

### Effect of noise correction

**Figure 2:**
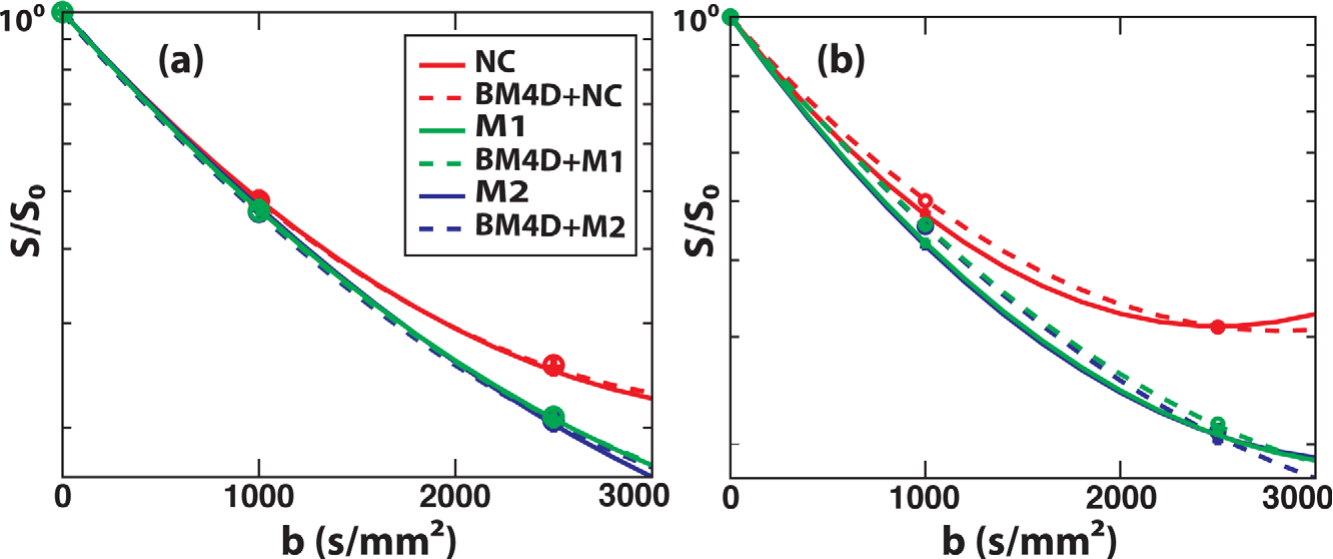
Normalized signal attenuation fits before and after noise corrections for the different correction schemes. The data correspond to a single diffusion direction in WM areas, for two different voxels with apparent SNR values equal to 26.3 (a) and 16.7 (b) respectively.

Figure 2 shows the effect of the different correction schemes on the signal decay for two voxels with significantly different SNR (26.3 and 16.7) in WM. The fitted values of *D*_app_ and *K*_app_ (Eq. (1)) for the different correction schemes are reported in Table 2. Correction has a stronger effect for higher *b*-values and/or for low SNR data points. As a result, the estimation of the apparent kurtosis *K*_app_ appears strongly affected by the noise bias: the difference between non-corrected and corrected values reaches about 25–30%. In contrast, the estimation of *D*_app_ is less strongly affected in all correction schemes (up to 10%). This is primarily due to significantly higher SNR and smaller noise bias observed for data points acquired at low diffusion weightings (*b* = 0 or 1000 s/mm^2^), which predominantly determine the estimated *D*_app_ value. The influence of BM4D filtering on *K_a_*_pp_ and *D*_app_ estimates appears negligible which is not surprising since the filtering procedure only reduces the local variance but does not correct for the noise bias.

**Table 2:** *K_app_* and *D_app_* (10^−3^ mm^2^/s) values for two voxels with different SNR, corresponding to Figure 1, with their standard errors.

	SNR = 26.3			SNR = 16.7
	K_app_	D_app_	K_app_	D_app_
NC	0.97±0.03	0.84±0.02	1.29±0.01	0.93±0.01
BM4D+NC	0.99±0.02	0.85±0.01	1.27±0.01	0.84±0.01
M1	0.74±0.03	0.85±0.01	0.89±0.01	1.00±0.01
BM4D+M1	0.76±0.02	0.87±0.01	0.84±0.01	0.90±0.01
M2	0.67±0.04	0.83±0.01	0.89±0.01	1.00±0.01
BM4D+M2	0.75±0.01	0.87±0.01	0.79±0.02	0.90±0.01

**Figure 3:**
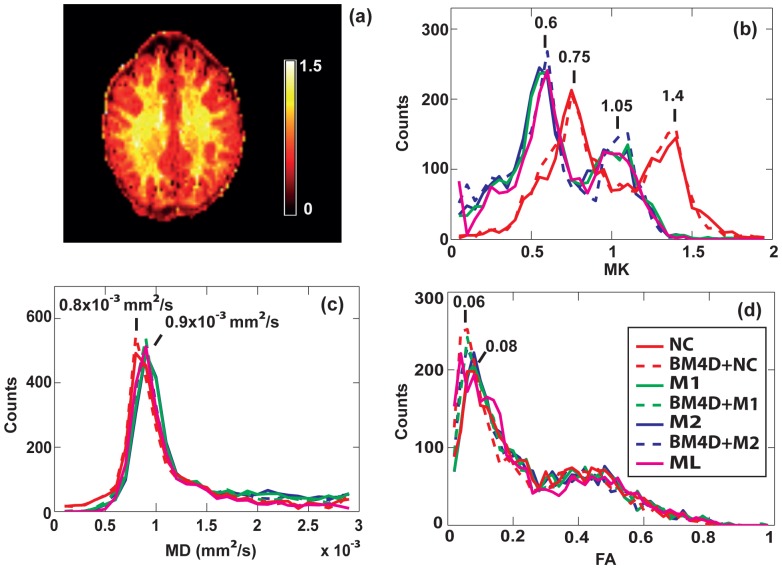
Histograms of diffusion parameters for one slice. (a) MK map for one single subject (position P1) and one selected slice corrected with method M1. (b) MK, (c) MD and (d) FA histograms of the same slice for uncorrected signal (NC and BM4D+NC) and using various correction schemes (M1, BM4D+M1, M2, BM4D+M2 and ML estimator).

**Figure 4:**
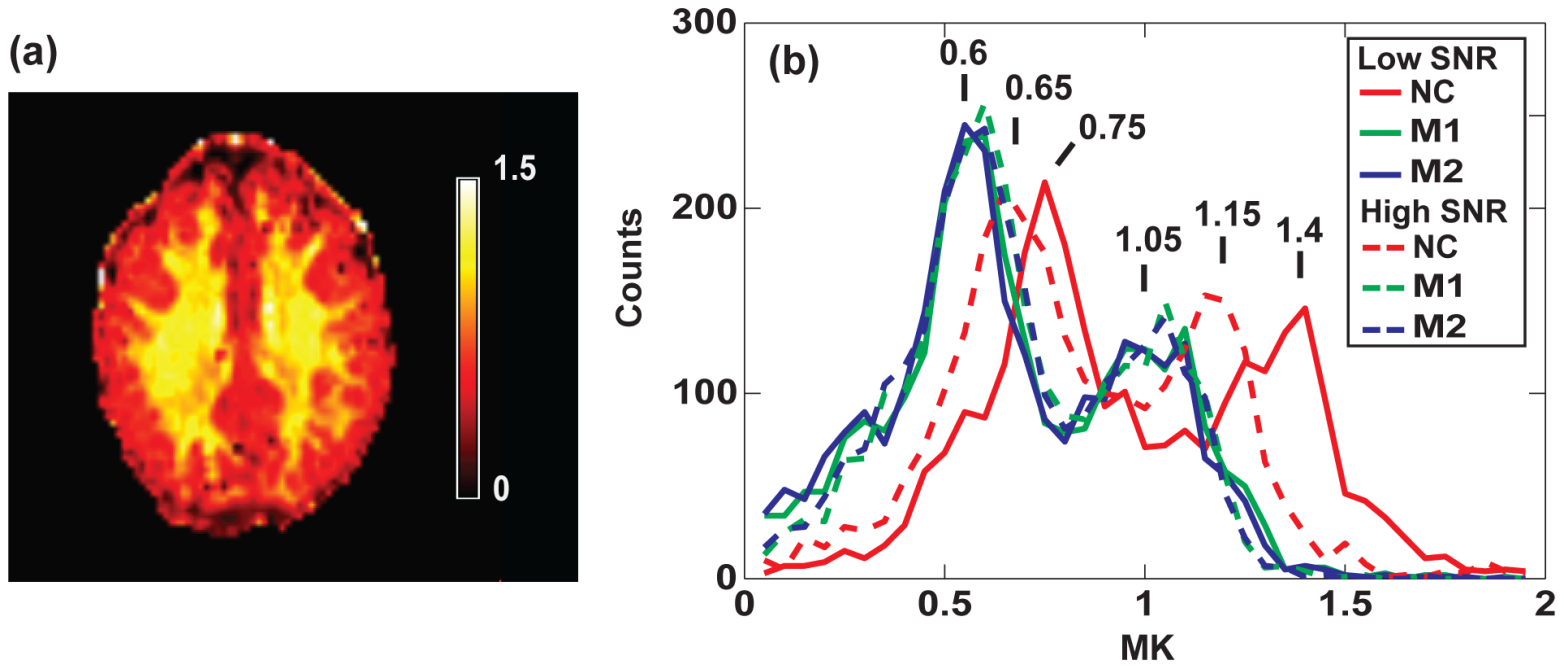
Map and histograms of MK for high SNR acquisition (P6). (a) MK map of the selected slice for high SNR acquisition (P6) corrected with M1 and (b) the MK histograms of the same slice for three correction schemes (dashed lines). The corresponding histograms for P1 (low SNR acquisition) are also shown (solid curves). The histograms are practically overlapping after the noise correction (blue and green curves). The non-corrected histograms are shifted with respect to each other. However, the difference between corrected and non-corrected values is smaller for higher SNR.

A map of MK obtained using the noise correction method M1 is shown in Fig. 3a, as an example. The corresponding histograms of MK, MD, and FA are shown in Figs. 3b, 3c, and 3d, respectively. In the absence of correction, MK values are strongly overestimated as demonstrated by the histograms in Fig. 3b. For both correction schemes, M1 and M2, the histograms exhibit significant shifts of both WM and GM peaks towards lower MK values as compared to non-corrected data: from 1.4 to 1.05 for WM and from 0.75 to 0.6 for GM. The two correction methods show similar results whereas BM4D filtering produces no significant effect. There are also no significant differences with the histograms obtained using ML estimator. Figure 4 compares Protocol 1a (P1) with low SNR acquisition and Protocol 1b (P6) with higher SNR acquisition for the same slice as in Fig. 3. After correction (M1) MK maps are similar for low and high SNR acquisition, as shown in Figs. 3a and 4a. The corresponding MK histograms for three corrections schemes (NC, M1 and M2) are shown in Fig. 4b. Similar histograms were obtained with corrections schemes using BM4D filter (not shown). The histogram of non-corrected data at high SNR (dashed red line) exhibits a clear shift compared to non-corrected data at lower SNR (solid red line) towards lower MK values. Moreover, the difference between non-corrected and corrected data becomes smaller at high SNR. In accordance with the analysis of the signal decay in Fig. 2, the impact of the noise correction on the estimation of MD and FA derived from the diffusion tensor is practically negligible, as demonstrated by their histograms in Figs. 3c and 3d. A slight shift towards higher mean diffusivity is observed in the MD histograms, since noise correction generally tends to increase the slope of the DW signal decay. The fractional anisotropy is almost unaffected, indicating a much smaller bias in estimated FA maps when no noise correction is applied.

### Intra-subject variability

**Figure 5:**
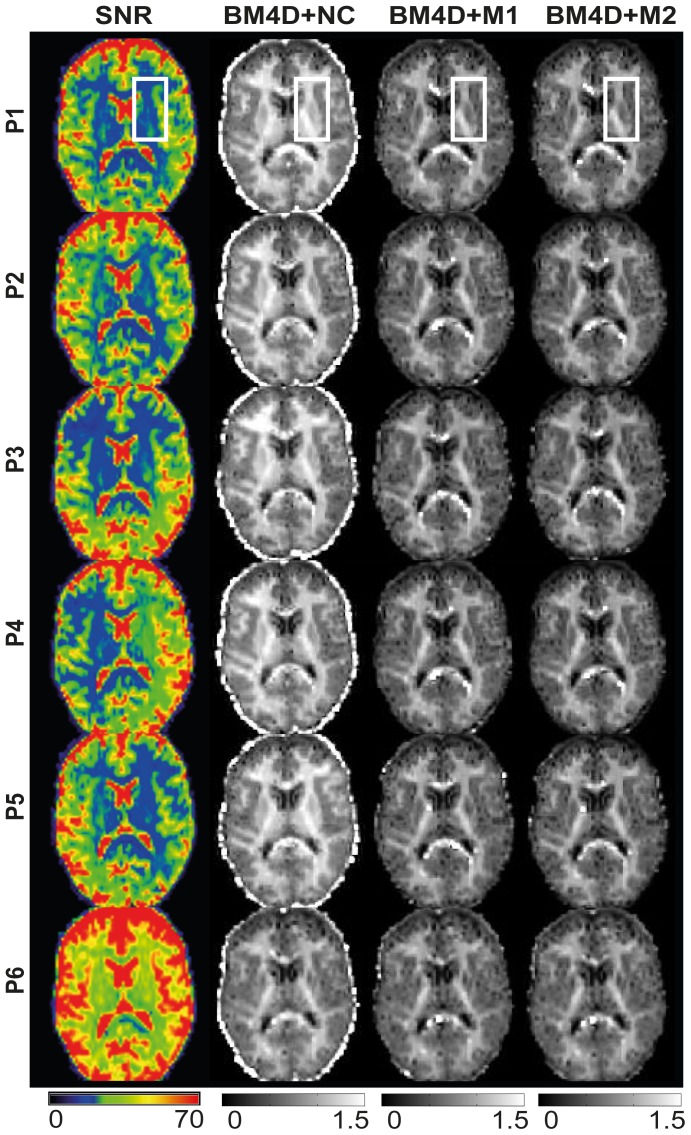
SNR and corresponding MK maps for low (P1 to P5) and high (P6) SNR acquisitions. The first column corresponds to SNR maps calculated as the ratio of the signal in non-DW images and the noise standard deviation. MK maps are shown in the other columns. Each row represents one acquisition with a different SNR profile. Results are shown for non-corrected (BM4D+NC) and corrected (BM4D+M1 and BM4D+M2) data. Results without BM4D filter are similar. The region delineated by a white rectangle is zoomed in Figure 6.

**Figure 6:**
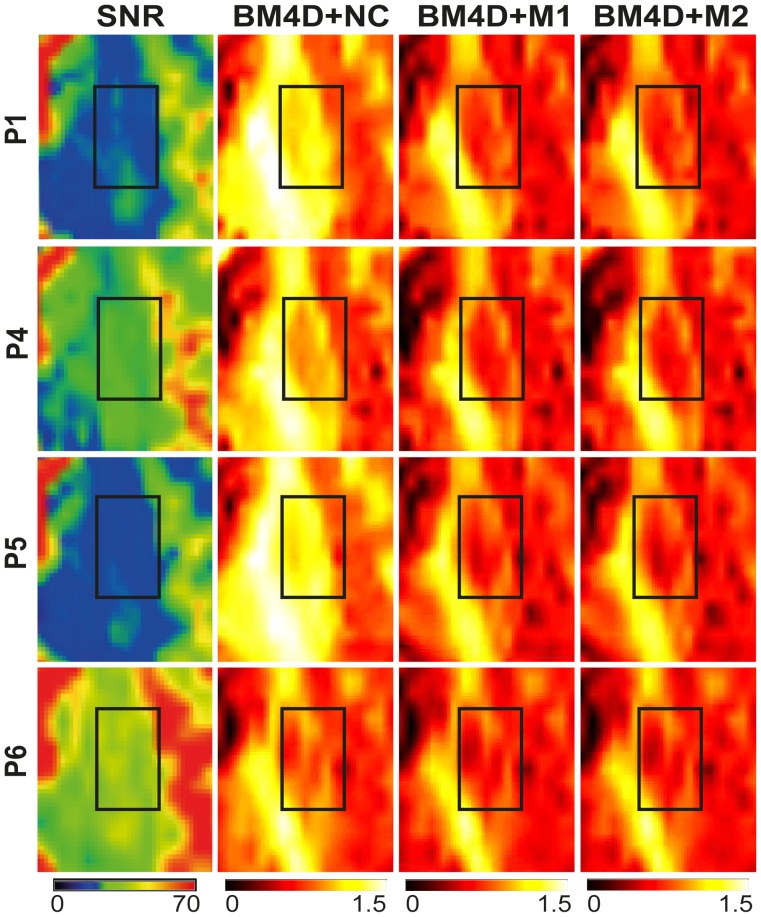
Zoom of SNR and MK maps for different SNR acquisitions. This figure corresponds to a zoomed area of Figure 5 for 4 selected positions (P1, P4, P5 and P6) and the same correction schemes (shown in colour for better visualization).

**Figure 7:**
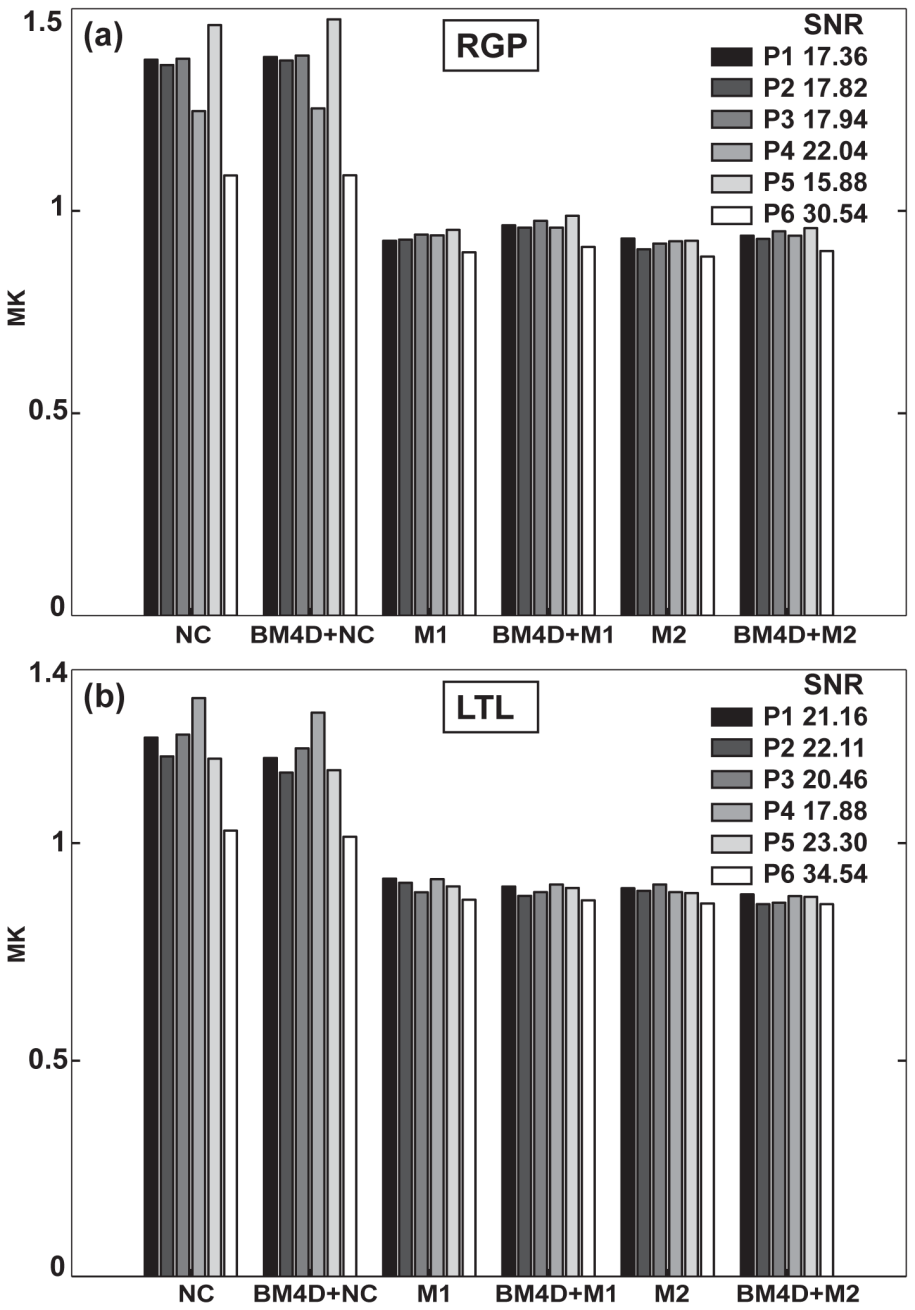
Averaged MK values for the different correction schemes in two different ROIs. (a) Right Globus Pallidus and (b) Left Temporal Lobe. Results are shown for the same subject with 6 different levels of SNR corresponding to acquisitions P1 to P6.

**Figure 8:**
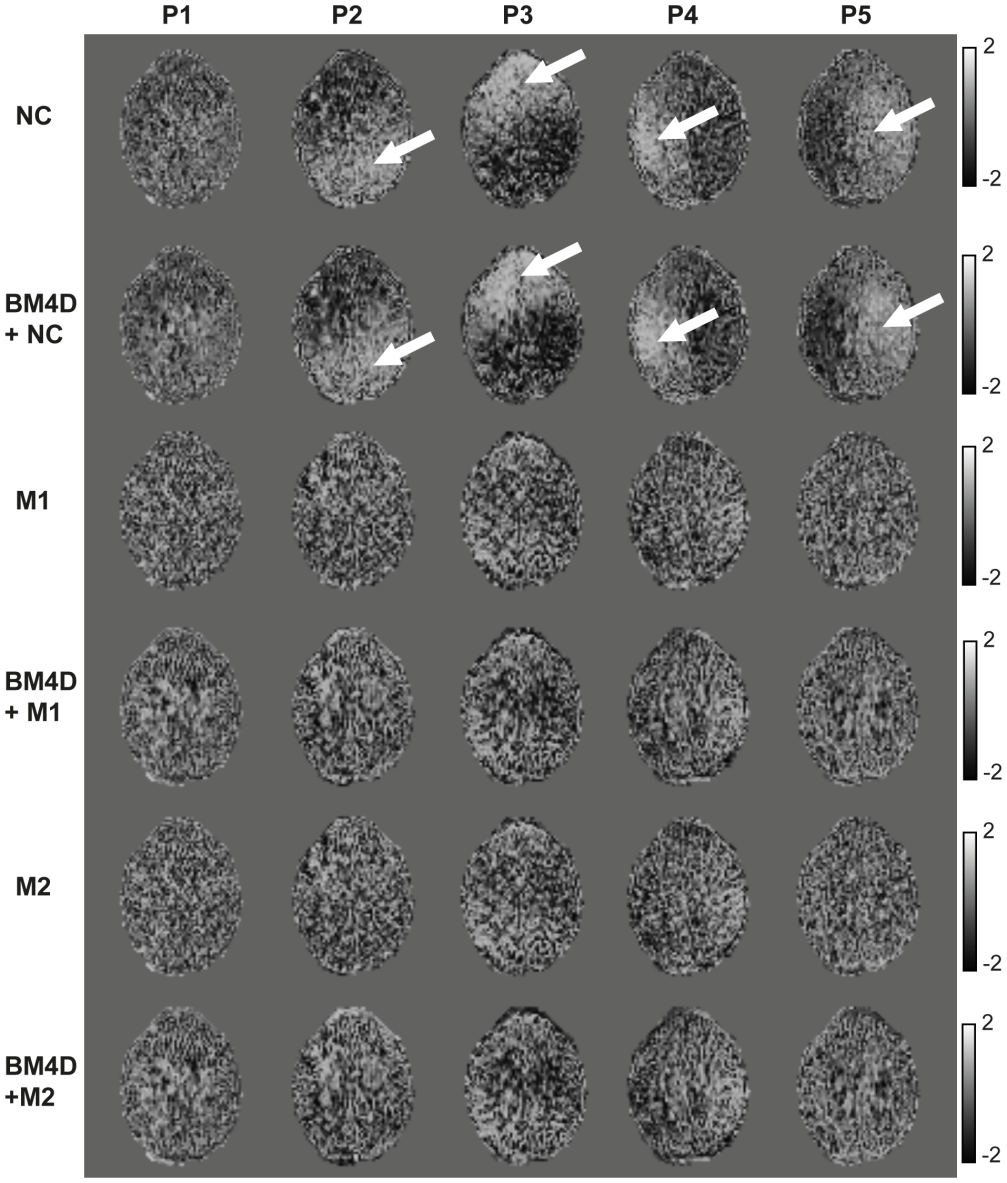
Z-score maps for each correction scheme (rows) and low SNR positions (P1 to P5). The reference is the average MK map over the 5 positions for each correction schemes. Arrows indicate regions of positive bias of MK due to lower SNR.

Results from the experiment 1 are shown in Figs. 5, 6, 7 and 8. In this experiment, the same measurement protocol was repeated 5 times for various positions of the head of the subject in the coil. As a result, the spatial distribution of SNR across the head was different in each of these measurements (P1 to P5), as illustrated in Fig. 5. The lower spatial resolution in P6 leads to a global 2-fold increase of SNR. In Figures 5 and 6, we compared the MK maps obtained after BM4D filtering and with and without noise correction. MK maps obtained without BM4D filtering were similar and therefore not shown. In the absence of noise correction, the MK estimate is systematically higher and depends significantly on the SNR. For example, the maps obtained at lower SNR (P1 to P5) exhibit higher values than the one obtained at higher SNR (P6). Besides, we observe that the regions with enhanced MK values correlate with lower SNR regions depending on the head position. This effect is further illustrated in Fig. 6, by a closer view of the region delineated in Fig. 5, for experiments P1, P4, P5 and P6. In that region, the SNR (and the MK estimates accordingly) varies strongly from one acquisition to the next when no correction is applied (Fig. 6, second column). The regions of lower SNR exhibit higher MK values. This effect is particularly marked in the region delineated by the white rectangle (Fig. 6). M1 and M2 corrections lead to very similar MK maps.

The influence of noise correction on the evaluated metrics was quantitatively assessed by statistical analysis in 8 ROIs. Examples are represented in Fig. 7 for the right Globus Pallidus and left Temporal Lobe where the MK values averaged over the indicated ROIs are compared for all correction schemes (see different bar groups) and positions (see bars within a given group). The averaged SNR values of the non-DW images corresponding to each session are indicated on the plots. In all sessions, non-corrected MK values remain higher than corrected ones. One can observe also that higher MK values correlate with low SNR values when no correction is applied. For example, in the left Temporal Lobe (Fig. 7b), lower SNR of 17.88 is associated with significantly higher MK of 1.34 in comparison to the value of 1.19 evaluated at higher SNR of 23.30, that is a significant increase of 13%. In contrast, when noise correction is applied, the MK estimate is globally lower and no longer dependent on SNR. For example, practically the same MK values of 0.92 and 0.9 were obtained for SNR values of 17.8 and 23.3, respectively. Moreover, Fig. 6 shows that the influence of noise is reduced at high SNR (e.g. SNR  = 34.53 in P6) where the difference between MK values estimated with and without noise correction exhibits an increase of about 16% while corresponding values obtained in sessions P1-P5 exhibits differences up to 50%.

The results obtained for different ROIs are summarized in Table 3, where the stars indicate the results of the Pearson's correlation tests. Generally, kurtosis values depend on the tissue (WM or GM) and can vary within an area consisting of a given tissue type. The mean MK over the ROI for each position (P1 to P5) is significantly correlated to SNR for non-corrected data (p<0.01) for all investigated ROIs. On the contrary, no correlations are reported for corrected data, except for two cases where a weak correlation is observed (p<0.05). The z-score maps shown in Fig. 8 emphasize this effect at the voxel level. Z-score maps indicate the positive or negative deviation of individual MK maps from the average MK map over protocols P1 to P5 for a given noise correction scheme, in units of the standard deviation. When no correction is applied, the z-score maps exhibit strong spatial heterogeneity (white arrows) in contrast to the homogeneous appearance of the z-score maps after correction.

**Table 3:** Mean MK values and standard deviations for each correction scheme and each ROI. P-values for Pearson correlation with SNR are indicated by: * p<0.05 and ** p<0.01.

	NC	BM4D+NC	M1	BM4D+M1	M2	BM4D+M2
**Right TL**	1.25±0.06**	1.21±0.06**	0.90±0.01	0.90±0.01	0.90±0.01	0.87±0.01
**Right GP**	1.36±0.06**	1.37±0.06**	0.92±0.02*	0.92±0.02	0.88±0.01*	0.90±0.02
**Right IC**	1.27±0.03*	1.25±0.04*	0.97±0.01	0.93±0.01	0.94±0.01	0.91±0.01
**Right ACR**	1.35±0.08**	1.34±0.08**	0.99±0.01	0.99±0.01	0.97±0.01	0.96±0.01
**Left TL**	1.18±0.08**	1.15±0.07**	0.89±0.02	0.88±0.02	0.87±0.02	0.86±0.02
**Left GP**	1.36±0.08**	1.37±0.08**	0.94±0.01	0.97±0.01	0.91±0.01	0.95±0.01
**Left IC**	1.27±0.05**	1.25±0.05**	0.97±0.01	0.95±0.01	0.96±0.01	0.93±0.01
**Left ACR**	1.31±0.06**	1.29±0.06**	0.97±0.01	0.97±0.02	0.95±0.02	0.95±0.02

All correlations were negative.

### Inter-subject variability

**Figure 9:**
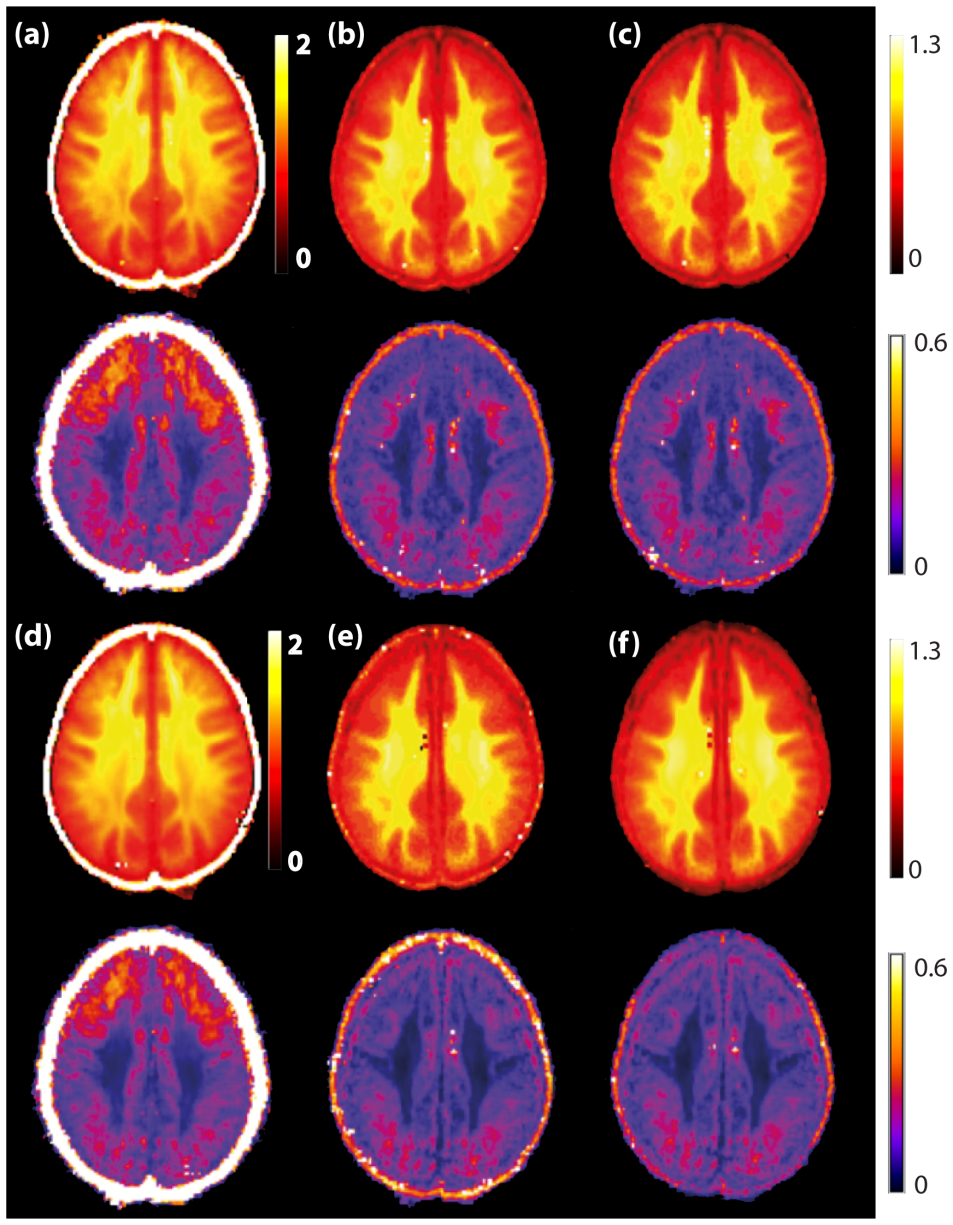
Averaged MK maps over 25 subjects and their standard deviation for one selected slice. MK maps are shown on first and third rows and the corresponding standard deviation maps on second and fourth rows for (a) NC, (b) M1, (c) M2, (d) BM4D+NC, (e) BM4D+M1, (f) BM4D+M2. (g) Mean SNR maps of the averaged non-DW image.

**Figure 10:**
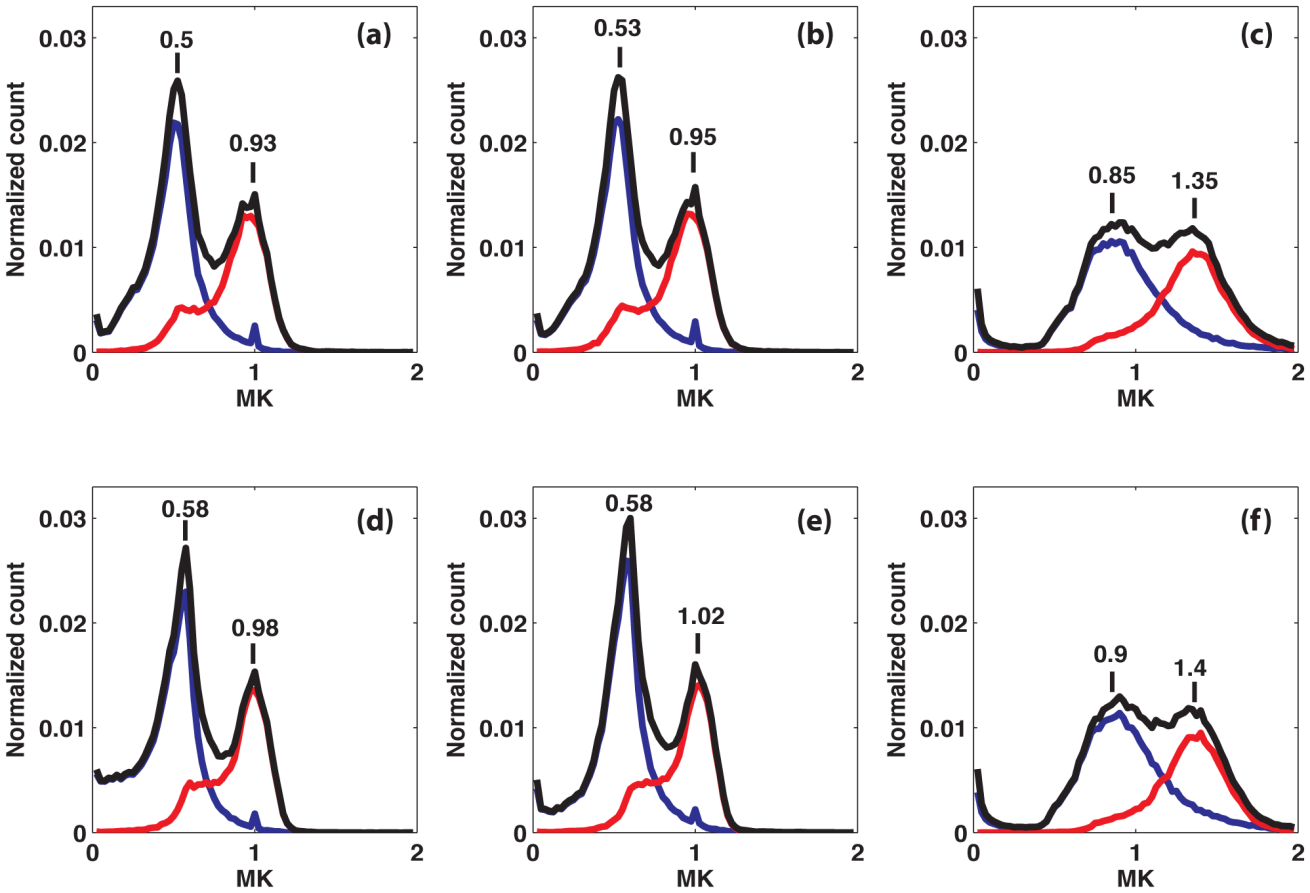
Histograms of the MK maps averaged over all subjects. The averaged histograms are shown for one selected slice (the same as in Figure 9) for the different correction schemes: (a) M1, (b) M2, (c) NC, (d) BM4D+M1, (e) BM4D+M2, (f) BM4D+NC. Black curves represent the whole slice histograms; red and blue curves refer to WM and GM parts, respectively.

In this section we examine the influence of noise correction on inter-subject variability and on the contrast between WM and GM in MK maps. As an example, Fig. 9 shows MK maps of one selected slice (at the level of Corpus Callosum) averaged over 25 subjects (first and third rows) and the corresponding standard deviations across all subjects (second and fourth rows). As shown in the previous section on a single subject, the MK is globally lower and the contrast-to-noise ratio (CNR) between WM and GM is higher with noise bias corrected data (Figs. 9b, 9c, 9e and 9f) as compared to non-corrected data (Figs. 9a and 9d). Quantitatively, these results are illustrated by the histograms in Fig. 10. After noise correction, the peaks of MK distribution are at 0.5 and 1.0, for GM and WM respectively, whereas, without noise correction, they are at 0.9 and 1.4 for GM and WM respectively. After correction, the peaks are significantly sharper and better separated, providing a better distinction between GM and WM. The effect of the BM4D filter is very subtle, leading to a slight increase of MK (+0.05).

Inter-subject variability is investigated through the standard deviation maps of MK across the group in Figs. 9a–9f (second rows) and the mean SNR map of the non-diffusion weighted image over the 25 subjects (Fig. 9g). On the one hand, standard deviation maps show that non-corrected maps are much more heterogeneous than corrected ones. The standard deviations reach a value of 0.4 in the frontal area that is twice as large as a value of 0.2 in the occipital area. On the other hand, on the mean SNR map, we observe a spatial gradient of SNR from top to the bottom, that is, SNR tends to increase towards the bottom of the image. This effect is related to hardware properties, more precisely to the spatially varying sensitivity of the coil array. When comparing both standard deviation maps and SNR maps, one can infer that the enhancement of standard deviation in the upper regions of the non-corrected maps correlates with observed SNR gradient from top to bottom. This result provides evidence that higher inter-subject variability of non-corrected MK maps is, in part, due to hardware-related SNR heterogeneity, and not only a genuine inter-subject variability. These results are supported by the ROI analysis averaged over 25 subjects. Numerical values of the MK are summarized in Table 4. In all ROIs and in the absence of noise correction, the MK averaged over all subjects is globally higher (by as much as about 50%) in comparison to the corrected value. The standard deviation of the MK is also systematically higher if no correction is applied.

**Table 4:** Mean MK values and standard deviation over 25 subjects for each correction scheme.

	NC	BM4D+NC	M1	BM4D+M1	M2	BM4D+M2
**Right TL**	1.36±0.07	1.34±0.07	0.84±0.05	0.85±0.03	0.83±0.05	0.81±0.04
**Right GP**	1.85±0.17	1.87±0.18	0.81±0.04	0.92±0.05	0.78±0.03	0.85±0.05
**Right IC**	1.49±0.07	1.87±0.18	0.96±0.04	0.94±0.02	0.94±0.03	0.90±0.02
**Right ACR**	1.49±0.07	1.48±0.06	0.90±0.04	0.95±0.04	0.89±0.04	0.91±0.04
**Left TL**	1.30±0.08	1.28±0.07	0.86±0.04	0.88±0.06	0.86±0.05	0.84±0.03
**Left GP**	1.76±0.15	1.79±0.17	0.81±0.07	0.92±0.04	0.79±0.05	0.86±0.04
**Left IC**	1.45±0.06	1.44±0.05	0.96±0.03	0.97±0.02	0.96±0.02	0.93±0.02
**Left ACR**	1.41±0.07	1.41±0.07	0.89±0.04	0.95±0.04	0.89±0.04	0.91±0.04

## Discussion

DKI has become a popular model for DW MRI and is often used as an advanced extension of DTI. However, this model suffers from low SNR at high *b*-values. For clinical applications where the acquisition time is a general issue and makes repeated measures for an increased SNR impossible, it is crucial to guarantee a good reproducibility and accuracy of the results at typically rather low SNR levels. In this paper, we considered two noise correction approaches and compared their performance under different SNR in terms of reproducibility of DTI and DKI metrics, such as FA, MD and MK, at both individual and group levels. We demonstrated the importance of these corrections for the reproducibility of the MK estimation, which then becomes independent of the SNR level. Classical DTI parameters were much less influenced. This is explained by the fact that the apparent diffusion coefficient estimation is mostly based on the lowest *b*-value data points where the SNR is higher and the noise correction has a smaller effect.

### Comparison of the different correction schemes

In a first experiment with simulated phantom data, two approaches for noise correction have been tested, one based on the analytical expression of the first moment of the noncentral chi distribution (M1) and the other based on the second moment (M2). These methods were compared with ML estimator, also accounting for the noncentral chi distributed noise, which have been shown to be more accurate than non-linear approaches [27], [29]. We showed that our methods and ML estimator gave similar results. This was also confirmed in one real data set. For the other experiments, we proceeded only with M1 and M2, in respect to time issues.

In addition, these corrections have been implemented with or without the non-local mean filter (BM4D). No significant differences between these methods have been detected in terms of parameters characterizing variability and reproducibility. This was confirmed by the simulations. However, by considering the number of brain voxels (including WM, GM and CSF) under the noise floor and thus forced to zero during the noise correction procedure, small differences were observed that can indicate variations in the robustness of the respective procedures. The non-local mean filter was expected to provide a more robust estimate of the first moment of the noncentral chi distribution. When applied, especially to higher *b*-value images, the number of voxels forced to zero during the M1 correction were reduced from 10% down to 2%, and from 11% to 3% during the M2 correction, indicating that a number of voxels with intensity below the noise floor have been correctly assigned to a value equal or slightly above the noise floor after filtering, which was the expected effect of the BM4D filter. This was confirmed by visual inspection of filtered versus non-filtered images (not shown). This result is slightly improved with M1 as compared to M2 (2% compared to 3%). M2 is a straightforward and easy to implement method. However, the squared data might amplify potential errors. M1 with BM4D filtering is therefore a preferred and recommended option.

### MK variability

Reported MK values in the literature are very inhomogeneous. In WM, mean MK values ranging from 0.51 in children [18] to 1.08 [53], ≈1 [22], 1.15 [23], and 1.39 [54] in adults, have been reported. In GM, the same studies reported MK values from 0.37 [18] to 0.73 [53] and 0.6 [23]. Correlation of MK with age for both WM and GM have been showed by [55], reporting values from 0.7 to 0.82 in GM and from 1.04 to 1.20 in WM. Noise bias correction was only applied in one of these studies [53]. These results are summarized in Table 5 for comparison. Our results after correction fall into this range, as we found WM peak for MK = 0.98 and GM peak for MK = 0.58 (M1+BM4D correction), for averaged data over 25 subjects. The discrepancies reported in the literature within the same age groups can be explained by the use of different approaches for image acquisition and data processing, and in particular by differences in SNR levels, which usually are not explicitly indicated. In order to reduce these differences and provide more robust and reproducible DKI estimates, including noise correction as a standard processing step, would certainly be beneficial. In particular, we have shown that two different acquisitions, with different SNRs due to increased voxel size (2.4 mm isotropic for the first one and 3 mm isotropic for the second one) give significantly different MK estimates when no correction is applied. However, this difference becomes non significant after correction. Noise correction methods, thus, allow the reproducibility and accuracy of the results at lower SNR. Acquisition at higher spatial resolution is thus clinically feasible without increasing acquisition time: partial volume effects are reduced and the chances to find small differences between two groups will increase.

**Table 5:** Summary of selected parameters from different published studies used for comparison.

Ref	*b*-value (s/mm^2^)	Number of directions	Age of the participants	Gender	Key regions	MK in WM	MK in GM
[18]	0/1250/2500	25	5–9	5M 3F (Healthy controls)	Temporal lobe	0.52±0.02	0.37±0.02
[53]	0/500/…/7000	6	55–56	11M 3F	Peak value over a slice	1.08	0.73
[22]	0/500/1000/2500/2750	15	36±13	12M 19F	Cingulum, corticospinal tract and corpus callosum	0.94–1.17	-
[23]	0/500/1000/2500/2750	15	33.1±12.5	16M 20F	Mean over all areas	≈1.15	≈0.6
[54]	0/500/…/2000/2500	30	36.5±12.3	9M 5F	Internal capsule	1.39±0.02	-
[55]	0/500/…/2000/2500	30	12–17;26–47;63–85	-	Prefrontal brain	1.04–1.20	0.7–0.82

All experiments were conducted at 3T.

The effect of noise correction was also shown by the ROI analysis in the selected regions, such as the Globus Pallidus. This region in particular is considered to be a good indicator of the SNR-related distortions in DKI analysis [10]. MK values in this region should be close to MK values in GM. However at low SNR, the combination of the noise bias effect and the comparatively short transverse relaxation time might result in a significantly elevated MK value. This “enhancement” effect was clearly demonstrated in Experiment 2 involving 25 subjects. MK values in the left and right GP are extremely high when data are not corrected (respectively 1.85 and 1.76 for NC) with a high inter-subject variability, and reach values close to GM areas for corrected data (respectively 0.81 and 0.81 for M1).

We have shown that the noise contribution varies with the acquisition protocol and can influence the total inter-subject variability. We have also shown that this variability is spatially variable and can be influenced by the position of the head in the scanner for example. However, after correction, no regional differences were found in terms of variability. The impact of variability of DKI parameters on study design and statistical power has been studied recently by [22]. In this work, they suggested that increasing the number of subject(s) will reduce the variability and is more beneficial than increasing scan time to gain SNR. However, by doing so, the total variability might be reduced but the noise bias is still remaining, leading to erroneous estimates. Reducing variability due to noise is thus very important and noise correction is highly recommended to get better estimates and derive reliable inference in group analysis.

### Applicability to clinical studies

In terms of group analysis, although only few clinical studies have been done yet with DKI, promising results have been showed. For example, DKI has proven to be a good marker for Parkinson's disease with an increase of 10 to 20% of MK values in the patient group as compared to the control group in the Caudate, the Globus Pallidus, the Putamen and the Substancia Nigra [56]. MK has also been shown to increase with higher tumor grades [57] with a minimum of 30% difference in MK values of different grades. Recently, DKI has been shown to be a good potential biomarker for Alzheimer's disease [58], [59]. In animal studies, significant differences, however, no larger than 10%, were reported, for example in the detection of Huntington disease [60]. Again, few of these studies included noise correction in their data processing and the SNR is not known. By decreasing the spurious inter-subject variability due to the noise bias, noise correction certainly will improve the statistical power of clinical studies, by allowing higher spatial resolution acquisition and smaller population samples.

### Limitations

The estimation of the noise standard deviation has been shown to be an important issue as it affects the subsequent noise correction procedure. Noise in SoS reconstructed magnitude images can be spatially varying as a result of the noise correlation between the channels of the receiver system [61]. In our study, the resulting spatial heterogeneity in the noise distribution had no noticeable effect due to the relatively small spatial variation of the noise standard deviation. The noise correlation was thus assumed to be of negligible impact on our noise procedure and the noise was considered spatially independent. As described in the methods section, the background noise distribution closely matches a noncentral chi distribution with a standard deviation that can be reliably estimated from Eq. (6), confirming the reliability of this assumption. In this work, spatially varying noise fields were thus not considered. Several factors like the use of parallel imaging and acceleration techniques [62] can increase the amplitude of noise spatial variations which then require more sophisticated methods of corrections [27], [63], already applied to DTI [64]. Developing similar methods for DKI will be the goal of future work.

Another potential source of bias in diffusion imaging is the physiological noise. The main effect of this noise is the appearance of artefacts close to the ventricles, which makes the estimation of diffusion and kurtosis parameter less accurate in those areas [26]. As modeling this type of noise and thus correcting for it a posteriori is very challenging, practical methods like cardiac gating have been shown to give better results [65], [66]. However, such methods increase acquisition time which is not convenient for clinical studies. Another solution is the application of robust statistics technique adapted to the kurtosis model of diffusion to detect and remove outliers due to physiological noise [35], [64]. However the investigation of such noise sources was beyond the scope of this research.

## Conclusion

We have proposed two noise correction approaches for DW images acquired with multichannel coils, with SoS reconstruction, and studied their impact on intra- and inter-subject variability in the context of DKI data analysis. Our results show that noise bias correction has a strong impact on MK estimation and that noise bias can lead to erroneous conclusions when conducting group studies. We demonstrated that the procedures described herein significantly reduce noise-related intra- and inter-subject variability and should not be neglected in DKI studies. Moreover, we provided a list of reference for kurtosis values in typical WM regions. As these regions were delineated using standard and available template, they can be easily reproduced by other groups for comparison. Evaluation including noise correction provides accurate and reproducible results independent of the SNR and the head position. Otherwise, the final MK maps are subject to biased errors depending on the spatial distribution of SNR caused both by differences in tissue relaxation and diffusion properties and, more crucially, by the spatially varying sensitivity characterizing multi-channel coils. The simplicity of the method described here allows a straightforward implementation and can be readily included in the regular pipeline for DKI analysis and the additional computational time is not much. Moreover, with such methods, the gain in reproducibility and accuracy of the results makes higher spatial resolution and lower SNR accessible, reducing partial volume effects in clinically feasible acquisition time. The statistical power is improved, increasing a confidence in the output results, or allowing one to reach significant conclusions with smaller population samples.
